# The role of the gut microbiota and metabolites in heart failure and possible implications for treatment

**DOI:** 10.1007/s10741-025-10546-7

**Published:** 2025-07-25

**Authors:** Ahmed Emad Ahmed Shoukry, Alaa Rahhal, Constantina Constantinou

**Affiliations:** 1 Department of Basic and Clinical Sciences, University of Nicosia Medical School, 21 Ilia Papakyriakou, 2414 Engomi, P.O. Box 24005, 1700 Nicosia, Cyprus; 2 Heart Hospital, Hamad Medical Corporation, Doha, Qatar

**Keywords:** Heart failure, Gut microbiota, Short-chain fatty acids, Trimethylamine, Trimethylamine N-oxide, Fecal transplantation

## Abstract

The prevalence of heart failure has increased significantly in recent years, prompting investigations into novel contributory factors. Among these, alterations in the gut microbiota composition have garnered attention due to their potential association with heart failure. Disruption in the bacterial environment associated with heart failure is characterized by heightened levels of *Proteobacteria* and *Firmicutes* and decreased levels of *Bifidobacteria* and *Bacteroides*. Reduced blood supply weakens the gut barrier, facilitating the transportation of bacteria and metabolites into the bloodstream. This breach can trigger an immune response and inflammation, subsequently contributing to the pathogenesis of heart failure through the generation of harmful organic compounds in the gastrointestinal tract and bloodstream. Specific metabolites, including short-chain fatty acids, trimethylamine, and trimethylamine N-oxide also contribute to the development of heart failure. Management of heart failure includes pharmacological management, surgery, and lifestyle modifications including recommendations for the consumption of a diet high in fruits and low in animal products. Heart failure can be managed by modulating the gut microbiota. Clinical interventions include antibiotics, prebiotics, and dietary changes. However, other approaches including fecal microbial transplantation, probiotics, and natural phytochemicals are still under study in animal models. This review highlights the significant yet underexplored link between gut microbiota and heart failure, suggesting that further research could lead to new therapeutic strategies and dietary recommendations to mitigate heart failure progression.

## Introduction

Heart failure is a clinical syndrome in which the heart’s ability to pump or fill with blood is reduced, resulting in inadequate circulation to meet the body’s demands. It often arises from underlying cardiovascular conditions that impair the structure or function of the myocardium, such as ischemic heart disease or hypertension, and can present in varied ways—from minor limitations during physical activities to severe symptoms at rest [1].

Different frameworks have emerged to categorize heart failure, each emphasizing a particular aspect of the condition. The New York Heart Association (NYHA) classification focuses on how much physical activity an individual can tolerate before symptoms appear, while the American College of Cardiology/American Heart Association (ACC/AHA) staging system addresses whether patients are at risk, have structural heart disease, or require advanced interventions. Another widely employed scheme identifies patients according to left ventricular ejection fraction, distinguishing between heart failure with reduced, preserved, or mildly reduced ejection fraction. Collectively, these classifications offer a structured way to evaluate disease progression and guide appropriate (Table 1) [2].

**Table 1:** The different heart failure classification systems: (1) Classification by New York Heart Association (NYHA), (2) Classification by American College of Cardiology (ACC)/American Heart Association (AHA) Stages, and (3) Classification by Left Ventricular Ejection Fraction (LVEF). These classifications are often used together to evaluate and manage heart failure. The three classification systems guide treatment decisions, monitoring, and counseling, based on the stage of disease, functional status, and underlying cardiac function. Clinical guidelines from organizations such as the ACC, AHA, and the European Society of Cardiology provide detailed recommendations on how to apply these classifications in clinical practice [2]

(1) Classification by New York Heart Association (NYHA) Functional Class	
Class I	Normal physical activity. Physical activity does not develop any symptoms such as breathlessness, fatigue or palpitations
Class II	Physical activity is slightly limited. Normal physical activity may result in breathlessness, fatigue, or palpitations. Patient is comfortable at rest
Class III	Physical activity results in marked physical activity restriction as symptoms of breathlessness, fatigue or palpitations occur earlier than usual. Comfortable at rest
Class IV	Any physical activity will result in discomfort. Symptoms may develop at rest as well
(2) Classification by American College of Cardiology (ACC)/American Heart Association (AHA) Stages	
Stage A	Patients at high risk for heart failure due to conditions such as hypertension or diabetes, but with no structural heart disease or symptoms yet
Stage B	Structural heart disease is present (e.g., reduced ejection fraction), but symptoms have not appeared
Stage C	Structural heart disease with current or past symptoms of heart failure, such as fatigue or shortness of breath
Stage D	Advanced disease where symptoms persist even with standard treatments; often involves considerations for specialized interventions
(3) Classification by Left Ventricular Ejection Fraction (LVEF)	
Heart failure with reduced ejection fraction (HFrEF)	LVEF ≤ 40%
Heart failure with preserved ejection fraction (HFpEF)	LVEF ≥ 50%
Heart failure with mildly reduced ejection fraction (HFmrEF)	LVEF of 41–49%

**Fig. 1:**
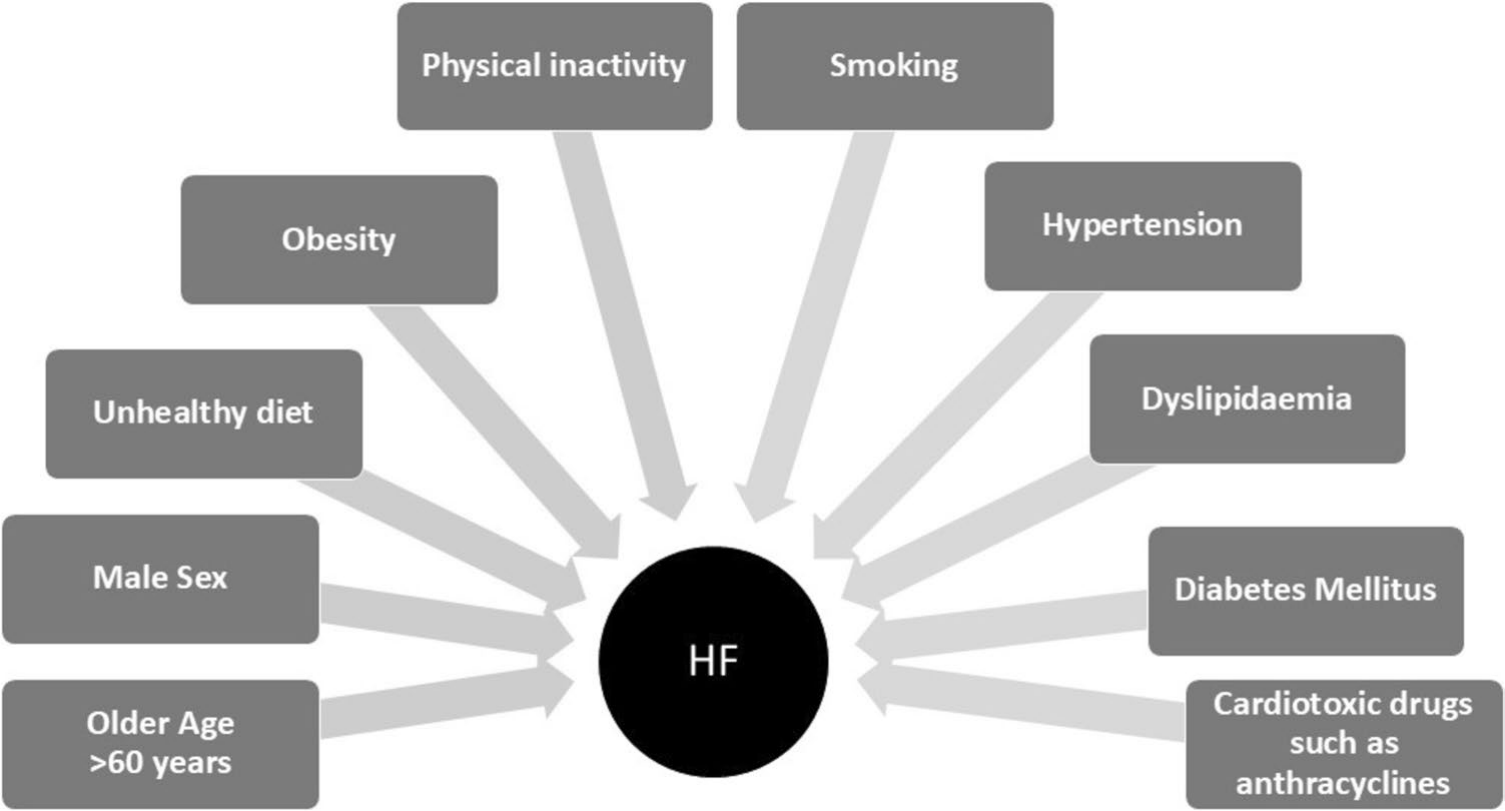
Risk factors associated with increased risk for HF (HF = heart failure) derived from McDonagh et al. (2021) [2]

According to the 2021 diagnosis and management guidelines from the European Society of Cardiology, multiple risk factors can contribute to the development of HF [2]. Common non-modifiable and modifiable risk factors of HF include older age (> 60 years), male sex, unhealthy diet, being overweight, physical inactivity, smoking, and suffering from hypertension or type 2 diabetes mellitus (Fig. 1) [3].

The human gut microbiota, initially formed from breast milk post-birth, establishes a symbiotic relationship with the host [4, 5]. The gut microbiota compromises of bacteria (representing up to 99% of all constituents) but also archaea, fungi, viruses, and protests [5]. The gut microbiota consists of elevated levels of *Bacteroides*, *Firmicutes*, *Actinobacteria*, *Proteobacteria*, and *Verrucomicrobia*, with *Firmicutes* and *Bacteroides* and *Firmicutes* constituting 90% of the total intestinal microflora. The latter can secrete metabolites, which can have hormone-like properties [6]. The microbiota balance and composition vary between individuals or organs in the same person based on genetic and environmental factors, including diet and lifestyle [6].

The gut microbiota plays a significant role in food digestion, nutrient absorption, energy generation, and the secretion of bioactive [6]. In addition, the gut microbiota is important for the formation and maintenance of the intestinal mucosal barrier, which is critical for preventing the proliferation of pathogenic microorganisms and supporting the maturation of immunological tissues [5].

Recent research has identified an association between HF and the gut microbiota (which includes bacteria and their metabolites). These metabolites, which include short-chain fatty acids (SCFAs) with hormone-like properties, bile acids, and trimethylamine N-oxide (TMAO) (a small organic molecule derived from trimethylamine (TMA)), can bind to host receptors and may serve as additional risk factors for HF [5–7]. TMAO, a small organic molecule derived from TMA, is formed enzymatically by gut microorganisms acting on dietary components, including red meat, fish, poultry, and eggs [7, 8]. Evidence indicates that TMAO contributes to atherosclerosis -a known risk factor for the development of HF- by promoting leukocyte adhesion to endothelial cells (EC) and activating NF-κB. Furthermore, TMAO reduces the ability of endothelial cells to repair themselves, which further predisposes to atherosclerosis, which, in turn, can progress into HF if coronary blood supply is sufficiently compromised [8].

The translocation of bacteria and their metabolites from the gut into the bloodstream, a process known as dysbiosis, has been implicated in the development of inflammation, further exacerbating the progression of HF [9]. Yet, despite emerging evidence, the mechanism through which the gut microbiota contributes to the development of HF remains unclear. Preliminary studies, such as the study conducted by Cui et al., report a microbial disequilibrium in the hearts of patients with Chronic HF (CHF), resulting in inflammation and malnutrition [6]. A theory known as “leaky gut” supports the hypothesis of gut dysbiosis and HF. Gut dysbiosis may result in either reduction of intestinal bacterial diversity or overproduction of harmful bacteria or loss of beneficial bacterial gut population [6].

Inflammation-induced by inflammatory markers is associated with cardiac fibrosis, a pathological process characterized by excessive and continuous deposition of extracellular matrix (ECM), collagen type-1, in the heart muscle. This leads to stiffening of the heart tissue, impairing its ability to contract and relax properly, and contributes to cardiac remodeling. The latter refers to the structural and functional changes in the heart in response to cardiac injuries or chronic diseases, further exacerbating the negative impact on heart function. Cardiac remodeling can lead to HF if left untreated. As a result, gut dysbiosis and HF interact in both directions since inflammation can exacerbate both conditions equally [6, 10, 11].

A potential connection between gut microbiota and heart failure (HF) has been proposed, as alterations in microbial populations appear to affect immune function and inflammation—two processes integral to HF development [6, 12, 13]. Most available data come from preclinical models showing that modifying gut microbial composition can reduce inflammation and improve cardiac function. Given that human studies investigating the association between the gut microbiota and HF remain limited, further clinical studies could clarify the molecular mediators and mechanisms by which the gut microbiota contributes to HF. A deeper understanding of these pathways may facilitate the design of therapies aimed at restoring a healthy microbiota, potentially mitigating HF progression or preventing its onset.

This review aims to assess the impact of gut microbiota on HF development, shedding light on potential avenues for future research and innovative therapeutic interventions.

## Methods

A literature search was conducted to gather relevant information on the association between heart failure and gut microbiota. We utilized three major databases: PubMed, Science Direct, and Springer. The search strategy employed a combination of keywords: “heart failure,” “gut microbiota,” “pathogenesis,” “short-chain fatty acids,” “trimethylamine,” “trimethylamine N-oxide,” “antibiotics,” “clinical guidelines,” and “treatment.” Boolean operators were used to refine the search results. The period for search spanned from 2013 to 2025. We restricted our selection to articles published in peer-reviewed journals and written in English. Additionally, authoritative sources such as the European Society of Cardiology, the American Heart Association Guidelines, and the Centers for Disease Control and Prevention were used to extract pertinent information. The guidelines used to extract information for management were the 2022 AHA/ACC/HFSA guideline for the management of HF and 2021 ESC guidelines for the diagnosis and treatment of acute and chronic HF.

## Regulation of the gut microbiome by endogenous and exogenous factors

The gut microbiota is influenced by both endogenous and exogenous factors. Beginning at birth, exogenous factors, including diet, bacterial infections, and the administration of drugs, can change the composition and diversity of the intestinal flora. Concurrently, endogenous factors, including genetic factors, humoral imbalance, weakened peristalsis, nutritional deficiency, acid–base imbalance, chronic intestinal congestion, or ischemic hypoxia, can potentially alter the composition of the gut microbiota [6]. Therefore, the dynamic interplay between genetics and environment acts as a dual influence in shaping the gut microbiota [14].

## Pathogenesis of heart failure and the gut microbiota

The gastrointestinal system plays an essential role in the pathogenesis of HF; the latter being referred to as “the gut hypothesis of HF” [6]. The gastrointestinal system, constituting 40% of the total human blood volume, is the initial organ to undergo diminished blood supply and the final organ to regain circulation, owing to its extensive vascular network. This renders it particularly susceptible to ischemic conditions, resulting in a state of hypoxia and hypercapnia [6]. Furthermore, reduced blood flow to the intestinal epithelium results in the transportation of sodium ions to the intestine through the sodium/hydrogen exchanger-3, leading to a low lumen pH [15]. These factors disrupt the gut microenvironment and alter the composition of the gut microbiota, contributing to a compromised intestinal barrier and increased permeability, commonly referred to “leaky gut” [6]. This phenomenon leads to systemic congestion, resulting in edema of the intestinal wall [15]. The compromised gut barrier increases gut permeability, facilitates the translocation of microorganisms, and allows the presence of bacteria, bacterial products, and microbial metabolites, such as lipopolysaccharides (LPS), in the systemic circulation [6].

Increasing evidence indicates that this altered gut flora and enhanced permeability can initiate chronic inflammation, resulting in plaque deposition and arterial blockage that reduces cardiac function. Individuals with bacterial DNA in their peripheral blood often produce elevated levels of inflammatory markers, such as interleukin (IL)−6 and C-reactive protein [15]. Moreover, the presence of LPS in bloodstream activates the NLRP3 inflammasome, a protein complex within the innate immune system that drives the production of IL-1β and IL-18, contributing to cardiac dysfunction [16]. Further research has shown that increased intestinal permeability exacerbates systemic inflammation, creating an endless cycle that negatively impacts both the gut and cardiovascular system. When the gut barrier is compromised, a greater number of bacteria and bacterial metabolites can translocate into the bloodstream. This triggers the host’s immune response, stimulating the release of pro-inflammatory cytokines such as TNF-α and IL-6. The resulting systemic inflammation contributes to cardiac remodeling and further deterioration of cardiac function, perpetuating the cycle of disease progression [16].

Pasini et al. [17] conducted a study to explore the association between HF and alterations in the gut microbiota. This study analyzed the bacteria in stool samples of participants (individuals with mild CHF, moderate to severe CHF, and control subjects). The findings revealed that patients with moderate to severe CHF exhibited increased intestinal permeability when compared to healthy controls. Notably, CHF patients had higher levels of pathogenic bacteria, including *Campylobacter, Shigella, Yersinina entercolitica*, and Salmonella compared to controls [6].

### Interaction between the gut microbiota and the host immune system

Under normal circumstances, the bacterium *Bacteroides fragilis,* which is commonly present in the gut flora, secretes polysaccharide A. This polysaccharide induces CD_4_^+^ T cells to convert into Foxp^3+^ Treg cells. Treg cells play a crucial role in controlling CD_4_^+^ T cell proliferation by preventing abnormal expression of T cell receptors, suppressing intestinal inflammation, and releasing anti-inflammatory cytokines such as IL-10 to maintain mucosal immune stability [6, 18]. Moreover, Treg cells can limit ventricular remodeling after infarction by reducing apoptosis of myocardial cells and myocardial fibrosis [6].

The host immune system is activated in response to elevated levels of pro-inflammatory cytokines, a key pathogenic factor in the development of HF. This is a consequence of the intricate interplay between mucosal immunity and gut flora. A crucial subtype of CD_4_^+^ helper T cells, Th17 cells, play a pivotal role in the body’s defense against bacterial or fungal infections. Th17 cells secrete inflammatory factors, including IL-21, IL-22, IL-17, and recruit neutrophils [6]. Experiments have demonstrated that humans have segmented filamentous bacteria (SFB), a group of commensal organisms genetically related to the genus *Clostridium*, located on the surface of the human intestinal epithelium as part of normal intestinal flora. The presence of SFB induces the production of innate and acquired immunity through the differentiation and maturation of Th17 in the intestinal tract [5, 19]. Specifically, these bacteria induce the expression of amyloid A, which, in turn, stimulates the secretion of IL-6 and IL-23 by dendritic cells in the lamina propria of the intestine, therefore contributing to the differentiation of Th17 cells. Studies have further established that IL-17 can contribute to the development of myocardial inflammation and myocardial ischemia–reperfusion injury [6]. Therefore, HF may be linked with the activation of CD_4_^+^ T cells, a significant contributor to cardiac remodeling and fibrosis [5].

### Τhe role of LPS in HF and its association with the immune system

**Fig. 2:**
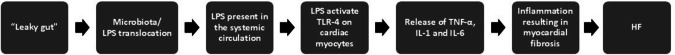
The association between gut microbiota and HF. Patients with HF have a “leaky gut,” which allows bacteria and released LPS to pass through the damaged intestinal mucosa and enter the systemic bloodstream. LPS activate toll-like receptors (specifically TLR-4) on cardiac myocytes, which release pro-inflammatory cytokines including TNF-α, IL-1, and IL-6, which lead to inflammation, myocardial fibrosis, and contribute to the development of HF (HF = heart failure, LPS = lipopolysaccharides) [4, 6, 14, 21–23]

Lipopolysaccharides (LPS) are a critical structural component of gram-negative bacteria [14]. In patients with HF, mucosal epithelial damage allows LPS to cross the compromised intestinal barrier and enter the systemic circulation. Once in the bloodstream, LPS interacts with toll-like receptors (TLRs), specifically TLR4, present on cardiac myocytes and endothelial cells. This interaction promotes the release of pro-inflammatory cytokines—such as IL-1, IL-6, and TNF-α [6, 14, 20], which have been found to be elevated in HF patients. These cytokines contribute to pathological changes in cardiac tissue, including hypertrophy, fibrosis, and apoptosis of cardiomyocytes [6, 14] (Fig. 2).

Additionally, LPS binding to TLR4 activates the nuclear factor kappa B (NF-κB) pathway, which further amplifies the inflammatory response by inducing the production of IL-1, IL-6, and TNF-α [4, 21–23]. Chronic release of these cytokines leads to persistent injury to cardiac tissue, progressively impairing myocardial function and promoting adverse cardiac remodeling, such as fibrosis and ventricular dilation. This remodeling worsens cardiac performance and accelerates the progression of HF [4, 21–23] (Fig. 2).

Prolonged exposure of cardiac myocytes to LPS and inflammatory cytokines can compromise their contractility. LPS disrupts nitric oxide (NO) signaling, resulting in elevated levels of cyclic guanosine monophosphate (cGMP). The increased cGMP then activates cGMP-dependent protein kinase, which further reduces contractile function. Consequently, cardiac output declines—a hallmark of HF [22].

Among the various inflammatory mediators released following LPS stimulation, three cytokines—TNF-α, IL-1, and IL-6—have shown particular importance in HF development and progression.

#### TNF-α

TNF-α may contribute to the development of HF by inducing apoptosis in cardiac myocytes and endothelial cells. Furthermore, TNF-α regulates the production of nitric oxide, which contributes to oxidative stress, and recruits neutrophils and macrophages. Studies have implicated TNF-α in the pathogenesis of angiogenesis and thrombogenesis, both of which are contributing factors to the development of heart disease [8].

#### IL-1

IL-1 signaling promotes the transcription of inflammatory genes, chemokines, pro-inflammatory cytokines, and adhesion molecules. Due to these mechanisms, inflammatory leukocytes are deposited in the injured cardiac tissue [24]. Moreover, IL-1 signaling plays a vital role in the pathogenesis of HF as it regulates the inflammatory process and increases the activity of matrix metalloproteinases (proteolytic enzymes that break down various proteins in the extracellular matrix). Consequently, IL-1 can suppress cardiac contractility, promote myocardial hypertrophy, and induce cardiac apoptosis [11, 19].

#### IL-6

It has been proposed that IL-6 contributes to the development of myocardial cell hypertrophy. Mice suffering from pressure overload–induced cardiac hypertrophy exhibited elevated levels of both IL-6 and IL-1 β [8]. IL-6, secreted from cardiac fibroblasts, contributes to cardiac hypertrophy through the p38 MAPK pathway, which regulates pro-inflammatory cytokine biosynthesis at the transcriptional and translational levels [8, 18].

Furthermore, prolonged exposure of cardiac myocytes to LPS and inflammatory cytokines may impair their ability to contract effectively. LPS reduces myocardial contractility by inactivating the nitric oxide (NO) signaling pathway, which leads to increased production of cyclic guanosine monophosphate (cGMP). Consequently, activated cGMP-dependent protein kinase in cardiac myocytes suppresses contractile function. As a result, cardiac output is reduced, a characteristic feature of HF [22].

### The role of SCFAs on HF

SCFAs, which include acetate, propionate, and butyrate, are key molecules produced in the gastrointestinal tract by gut bacteria. SCFAs maintain an intact gut barrier and influence cell growth, gut motility, and proliferation [4].

SCFAs are absorbed into the bloodstream by the portal vein and bind to receptors such as GPR109A, GPR43, and GPR41on gut epithelial and immune cells [9, 25]. SCFAs also modulate immune cell responses influencing local inflammation and regulating cytokine release—including IL-6 and IL-8 -by various leukocytes [4, 5].

Among SCFAs, butyrate binds to receptor GPR41, stimulating the expression of transcription factor hypoxia-inducible factor 1 alpha (HIF1-α). In addition, butyrate inhibits histone deacetylase (HDAC), leading to the acetylation of hormone response elements (HREs) on the promoter of cytokine interleukin-22 (IL-22). This acetylation promotes the binding of HIF1- α, resulting in the production of IL-22 CD4 + T cells and innate lymphoid cells [5]. IL-22 is essential in preserving gut integrity through its roles in mucus secretion, epithelial growth and permeability, and anti-microbial protein synthesis (AMPs), thus preventing intestinal inflammation. [5]. The inhibitory effect of HDAC may also help reduce cardiac dysfunction [4]. Collectively, SCFAs exert protective effects against HF as they stabilize myocardial metabolism, regulating inflammation and supporting the gut barrier, and their depletion can contribute to the development of HF [4, 5].

### The role of TMA and TMAO on HF

The gut flora, primarily consisting of *Firmicutes and Proteobacteria*, contributes to the production of trimethylamine (TMA) from digested foods. Once formed, most TMA is absorbed into the bloodstream and then oxidized into trimethylamine N-oxide (TMAO) by hepatic flavin-containing mono-oxygenase 3 (FMO3) [26, 27]. However, when the intestinal mucosal barrier is compromised and permeability increases, TMAO more readily enters the systemic circulation. bloodstream. Consequently, elevated levels of TMAO accumulate in organs such as the kidneys and heart, triggering processes including foam cell formation, platelet aggregation, and inflammatory responses. Over time, these disturbances adversely affect cardiovascular function and may culminate in HF [14].

Multiple studies indicate a significant link between elevated TMAO levels and HF, with the gut microflora emerging as a key determinant [6, 14]. TMAO specifically disrupts stimulus-dependent calcium signaling, increasing platelet reactivity, and elevating the risk of atherosclerosis and thrombosis. Additionally, TMAO activates NLRP3 inflammasome, resulting in the secretion of pro-inflammatory cytokines, such as IL-1β, IL-6, and TNF-α in human aortic endothelial cells and vascular smooth muscle cells (VSMCS) [9, 28]. TMAO also promotes leukocyte adhesion to endothelial cells, activating the p-38 MAPK pathway and NF-κB, processes that further heighten inflammation and endothelial dysfunction, which are critical contributors to the development of HF [6, 28].

Studies indicate that TMAO contributes to HF pathogenesis through several mechanisms. In mice models, TMAO directly activates the NF-κB pathway, prompting vascular smooth muscle cell inflammation and mitochondrial reactive oxygen species accumulation. TMAO also stimulates the NLRP3 inflammasome, which leads to endothelial cell inflammation and vascular calcification [14]. Additionally, TMAO disrupts mitochondrial function and intracellular calcium processing in cardiomyocytes, impairing contractility and promoting adverse ventricular remodeling [14]. TMAO further aggravates myocardial inflammation by increasing TNFα levels and reducing IL-10, fostering an environment conducive of myocardial fibrosis. This fibrotic response, driven in part by the Smad3 pathway within the transforming growth factor beta family, is marked by collagen deposition and tissue scarring [14, 29]. Elevated TMAO can also exacerbate renal dysfunction, causing sodium and water retention and interstitial fibrosis, cumulatively contributing to HF progression [14].

In summary, studies have consistently found an association between elevated TMAO and HF, indicating its potential as a marker for HF [9, 30].

### Controversies regarding the role of TMAO as a risk factor for HF

While elevated TMAO levels are commonly implicated in HF pathogenesis, certain studies suggest it may exert cardioprotective effects. For instance, chronic administration of low-dose TMAO in hypertensive rats led to significant reductions in plasma NT-proBNP and vasopressin levels, left ventricular diastolic pressure, and cardiac arrhythmias [31]. Similarly, Zhang et al. [14] reported an inverse relationship between the concentration of TMAO and the size of aortic lesions, proposing that TMAO may prevent the development of atherosclerosis. However, the mechanisms underlying these observations are not clear. Further research should explore both TMA and TMAO levels and their associations with HF, aiming to clarify their roles in cardiovascular diseases [14].

## Management of HF

**Fig. 3:**
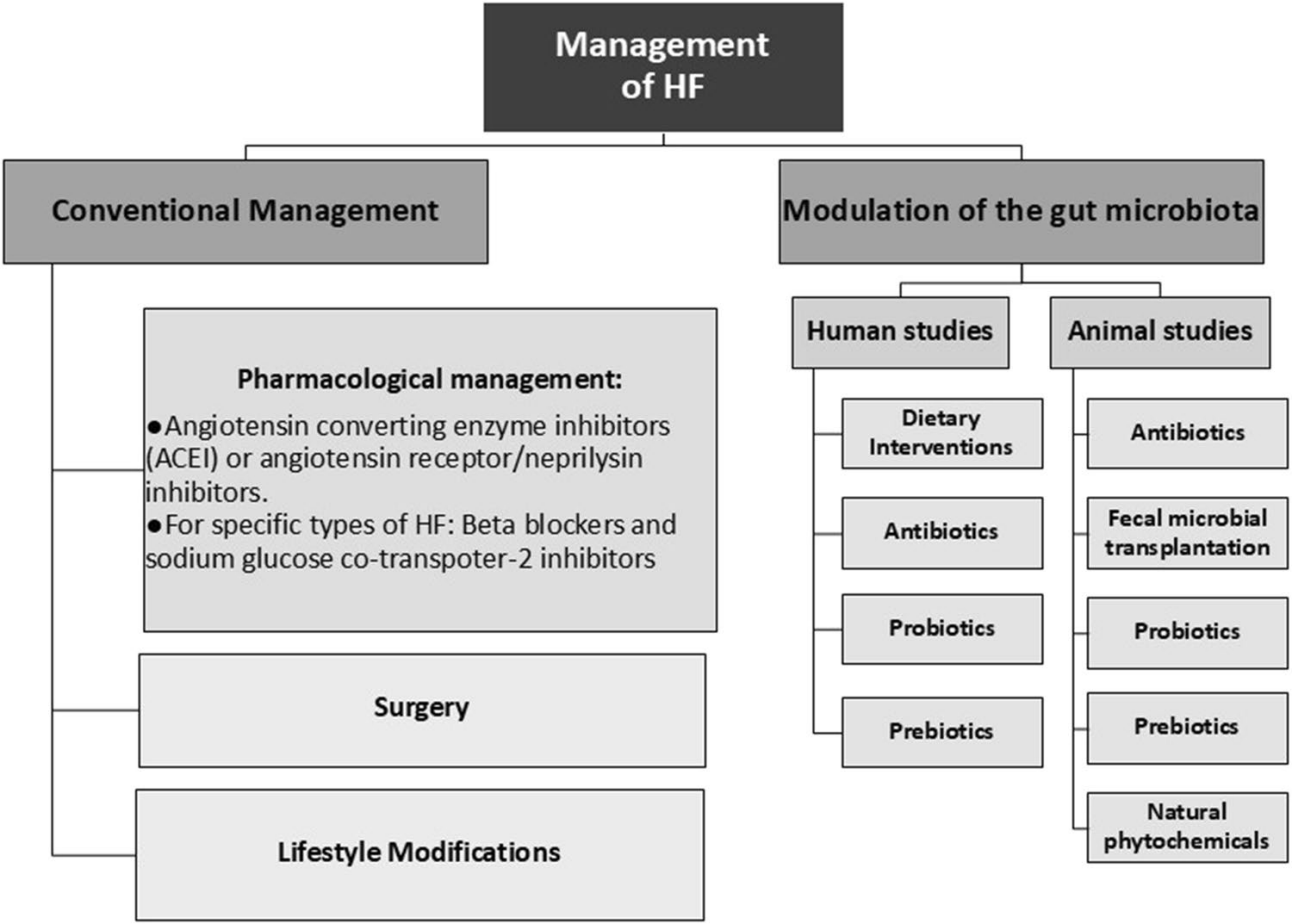
Conventional management of HF according to 2022 AHA/ACC/HFSA guidelines for the management of HF [2, 32, 33] and ongoing clinical trials and animal studies that aim to modulate the microbiota to improve gut flora aiming to restore hemostasis (HF = heart failure) [9, 14, 35, 38, 43, 45]

HF can be treated through established therapies following 2022 AHA/ACC/HFSA guidelines for the management of HF, while new strategies are continuously being investigated (Fig. 3).

### Conventional management of HF

The conventional management of HF is centered around pharmacological management, surgery, and lifestyle modifications [32].

#### Pharmacological management

Four primary classes of medications form the cornerstone of HF therapy: angiotensin-converting enzyme (ACE) inhibitors, beta-blockers, mineralocorticoid receptor antagonists (MRAs), and sodium-glucose cotransporter 2 (SGLT2) inhibitors [32].

##### ACE Inhibitors

ACE inhibitors are usually introduced first to lower mortality and morbidity, particularly in patients with HFrEF and New York Heart Association (NYHA) class II to III symptoms. If patients experience a dry cough or angioedema from ACE inhibitors, angiotensin receptor-neprilysin inhibitors (ARNIs) or angiotensin receptor blockers (ARBs) are recommended [32].

Beta-blockers (for example, bisoprolol) are added to decrease both mortality and hospitalization risk. Clinical trials indicate that beta-blockers significantly reduce cardiovascular events in individuals with HFrEF, making them a key component for all such patients [32]. An MRA is incorporated alongside ACE inhibitors and beta-blockers to further reduce mortality and morbidity, though close monitoring is advised for those at risk of renal impairment or hyperkalemia [32].

Patients with HFrEF and diabetes benefit from prompt initiation of SGLT2 inhibitors, which have shown additional cardiovascular and renal advantages [32]. Individuals of African American descent with NYHA class III–IV HFrEF are advised to add hydralazine plus isosorbide dinitrate if the standard triple therapy proves insufficient at improving symptoms or lowering morbidity and mortality [32].

#### Surgery

Individuals with advanced HF may require surgical interventions to address underlying causes [33]. Common procedures include coronary artery bypass grafting, surgical ventricular restoration, cardiac transplantation, cardiac support devices, and valve repair, with the choice of intervention depending on the etiology and severity of HF [33].

##### Left ventricular assist device (LVAD)

An LVAD is often considered for patients without significant right ventricular dysfunction who have severe left ventricular ejection fraction (LVEF < 25%) with marked exercise intolerance, multiple heart failure–related hospitalizations in the preceding year, or progressive end-organ dysfunction attributable to inadequate perfusion. Once an LVAD is placed, CABG is contraindicated [2, 32].

##### Coronary artery bypass grafting (CABG)

In patients with chronic coronary syndrome (CCS) and multivessel coronary artery disease, CABG is the preferred revascularization method. The procedure aims to enhance myocardial perfusion, alleviate ischemic burden, and improve overall cardiac function, particularly in HF patients [2].

##### Heart transplantation

For individuals with end-stage HF who do not respond to optimal medical therapy and need continuous inotropes or vasopressors to sustain cardiac output, heart transplantation is often considered. These patients typically experience profound cardiac reserve depletion and continued clinical deterioration despite standard treatments [2].

##### Valvular surgery

When valvular heart disease underlies HF, the specific approach depends on the affected valve and clinical presentation. For aortic valve dysfunction, transcatheter aortic valve implantation (TAVI) or surgical aortic valve replacement (SAVR) may be indicated, while mitral regurgitation often necessitates mitral valve repair or replacement based on disease severity, symptoms, and surgical risk [2].

#### Lifestyle modifications

Lifestyle modifications are integral to managing HF because they target modifiable risk factors and promote overall cardiovascular health. Smoking cessation remains critical, as smoking exacerbates endothelial dysfunction and can further compromise cardiac function. Engaging in regular physical activity—tailored to an individual’s functional capacity—supports cardiopulmonary fitness, alleviates symptoms, and helps sustain a healthy body weight. Adopting a low-salt diet reduces fluid retention and eases the strain on the heart by lowering blood pressure. Additional measures, such as moderating alcohol intake, controlling body weight through balanced nutrition, and monitoring fluid intake, can further optimize clinical outcomes and enhance quality of life for individuals with HF [2, 32].

### Clinical studies investigating the role of dietary interventions in the modulation of the gut microbiota and the management of HF

The gut microbiota is significantly influenced by dietary habits [14]. Long-term dietary patterns have a profound impact on the gut microbiota. A Western diet, characterized by high consumption of high animal protein, carbohydrates, and saturated fat, elevating plasma, and urine levels of TMAO, contributes to gut bacterial dysbiosis, and consequently increases the risk of cardiovascular disease [14].

In contrast, evidence suggests that the Mediterranean diet, which is rich in vegetables, fruits, whole grains, and nuts, and includes a limited intake of animal protein such as meat and eggs, promotes optimal gut flora status [14]. A high fiber diet results in lower plasma levels of TMAO thereby preventing HF development and alleviating ventricular remodeling. Additionally, extra-virgin olive oil, a key component of the Mediterranean diet, contains the organic substance 3,3-dimethyl-1-butanol (DMB), which plays a role in lowering TMAO levels by inhibiting TMA formation. Consequently, there is a notable link between the Mediterranean diet and the reduction in TMAO production [14].

Like the Mediterranean diet, the DASH diet emphasizes a high intake of fruits, vegetables, whole grains, and low animal protein. This diet is beneficial to HF patients as it is rich in fiber, micronutrients, and antioxidants. The DASH diet is associated with low reactive oxygen species, low pro-inflammatory cytokines, and efficient endothelial function, which contributes to a decreased incidence of HF. This eating pattern is also known for its ability to lower blood pressure as it is characterized by low sodium and high potassium content. Moreover, the DASH diet is high in magnesium, calcium, nitrates, and antioxidants, further contributing to cardiovascular benefits [9].

Research findings indicate that a brief dietary transition to the Mediterranean or DASH diet for just 5 days can significantly change gut microbiota composition. This adaptability to dietary changes and the subsequent reduction in the risk of HF incidence surpass the benefits of HF medications as per the guidelines [14].

There is weak evidence to support that the DASH diet could be implemented for managing HF patients [34]. One study provided evidence for an association between the DASH diet and improved cardiac function assessed by stroke volume and end-diastolic volume. A study conducted by Juraschek et al. [35] aimed to investigate the impact of the DASH and sodium-restricted diets, both individually and in combination, on biomarkers of cardiac injury, strain, and inflammation. These findings revealed that the DASH diet alone led to a decrease in the marker of cardiac injury high sensitivity cardiac troponin1 (hs-cTn1), while the sodium-restricted diet alone lowered a marker of cardiac strain NT-proBNP. A combined DASH and low sodium diet resulted in a significant decrease in both hs-cTn1 and NT-pro-BNP [35]. It is important to note that this study focused on a population following the DASH diet with untreated hypertension and no evidence of any CVD, and participants were provided with highly controlled study meals. However, there is considerable evidence to determine whether these mechanisms and biomarkers would be similarly effective in diagnosed HF patients, with and without preserved ejection fraction [34].

### Studies investigating the role of antibiotics in the modulation of the gut microbiota and the management of HF

Antibiotics can disrupt the composition of the normal intestinal flora. Studies initially conducted with animals, and subsequently with humans, have provided evidence that antibiotics play a role in preventing the pathogenesis of HF [36].

#### Animal studies

There is evidence to suggest that antibiotics can impact cardiac function. For instance, a study conducted on mice with myocardial infarction provided evidence that antibiotics such as vancomycin (a glycopeptide antibiotic that is effective against a range of Gram-positive bacteria) can prevent the translocation of bacteria through the gut barrier, thereby alleviating systemic inflammation and myocardial cell damage [30, 37].

#### Human studies

In a Phase II trial [38] designed to investigate the impact of antibiotics on gut microbiota, 150 stable individuals with HFrEF were divided into three groups, receiving either rifaximin, probiotic yeast (brady yeast), or no treatment at all. The primary endpoint was to measure the left ventricular ejection fraction (LVEF) after 3 months using echocardiography. LVEF assesses the pumping function of the heart’s left ventricle. Therefore, a lower LVEF indicates reduced pumping function, which can be a sign of heart disease or HF. The results of the stud demonstrated that when added to the standard of care treatment with rifaximin or probiotic yeast, it did not significantly affect LVEF and therefore did not improve cardiac function [38].

Zhang et al. [14] illustrated that short-term and low-absorbance antibiotics inhibit TMAO synthesis. In individuals with HF, Polymyxin B (an antibiotic that belongs to the polymyxin class of antibiotics and is primarily used to treat infections caused by Gram-negative bacteria) and Tobramycin (an aminoglycoside antibiotic use used to treat various types of bacterial infections, particularly those caused by Gram-negative bacteria) can lower LPS levels in the colon and feces and the levels of pro-inflammatory cytokines, including IL-1, IL-6, and TNF in humans. Furthermore, those antibiotics can improve endothelial function in HF patients [14].

Excessive use of antibiotics may harm humans since they can eliminate beneficial bacteria in the body and lead to drug-resistant microbiota in the gut [14]. Therefore, clinicians should weigh the benefits and risks of using antibiotics before using the medication. Moreover, extensive research may be conducted to evaluate whether the use of antibiotics in certain presentations will affect heart function and improve the HF survival rate.

### Studies investigating the role of fecal microbiota transport (FMT) in HF

FMT is a procedure for treating intestinal dysbiosis and re-creating healthy gut microflora by providing bacteria or donor feces metabolites into recipient individuals. The effectiveness of FMT as a potential intervention for HF is still under investigation [39].

FMT has demonstrated clinical efficacy in treating conditions such as infection with *Clostridium difficle* and inflammatory bowel disease [40]. Moreover, there is emerging evidence of a “super-donor” phenomenon where fecal samples from specific donors are more likely to achieve successful FMT outcomes than other donors. A random double-blind control on patients with metabolic syndrome concluded that vegetarian fecal flora after single transportation could alter the composition of patients’ intestinal flora but could not modify vasculitis-related parameters [6].

#### Animal studies

FMT is currently under investigation in animal studies. Studies have provided evidence that FMT may lower the levels of TMAO, whereas in other studies, there was overproduction of TMAO [14, 41]. A probable reason for the variability could be the number of bacteria transplanted, and therefore, further research should be conducted to determine the most appropriate levels of bacteria that should be used in FMT to achieve the appropriate decrease in TMAO [41].

Further studies should be performed in HF patients to address the current limitations of completed animal studies including concerns with infection and rejection. Furthermore, further research needs to be conducted to analyze the clinical significance of FMT in the field of cardio-metabolic disorders [14].

### Studies investigating the role of prebiotics and probiotics on the modulation of the gut microbiota and the management of HF

Recent literature has proposed that prebiotics and probiotics may regulate myocardial remodeling in HF patients.

#### Prebiotics

Prebiotics are a group of non-digestible foods, such as fibers, which stimulate the growth and activity of beneficial bacteria, thus contributing to a healthy gut [18, 28].

##### Animal studies

A study was conducted to investigate the effect of a prebiotic complex (fermented wheat bran and inactivated *Saccharomyces cerevisiae* culture) on the levels of endotoxin and microbial imbalance on rats suffering from HF. The results of the study showed that the use of prebiotics in rats suffering from HF decreased the levels of endotoxin [42]. Therefore, prebiotics may potentially restore the gut microbiota, reduce endotoxemia, and decrease inflammation [14].

##### Human studies

A randomized cross over trial (RCT) implemented on obese patients experimenting with the effect of inulin, a prebiotic, on overweight and obese adults [14]. The study concluded that insulin stimulates the production of SCFAs, reducing systemic inflammatory markers and improving insulin sensitivity. Insulin also contributes to the growth of beneficial bacteria and enhances the diversity and function of gut flora [14, 43]. Other studies also support the role of prebiotics in preventing low-density lipoprotein oxidation, lowering blood pressure, and preventing visceral obesity [14].

#### Probiotics

Probiotics are live microorganisms, including bacteria (like *Lactobacillus* and *Bifidobacteria*) and yeast, which can enhance healthy digestion and play a protective role in HF [9, 36].

##### Animal studies

A study on rats with HF who were administered with *Lactobacillus rhamnosus GR-1* showed significant decreases in left ventricular hypertrophy and apparent improvements in both systolic and diastolic hemodynamic parameters [14]. Furthermore, another study using mice conducted by Tang et al. [44] demonstrated a link between gut flora and cardiac function. It showed that administering probiotics before a myocardial infarction initiates cardioprotective effects, as the balance of SCFAs to propionic acid shifts and TMAO levels decrease [44]. In conclusion, although there is currently insufficient evidence to support the treatment of HF patients with probiotics, this treatment strategy holds excellent prospects [14].

##### Human studies

A randomized double-blind, placebo-controlled pilot trial was conducted by the New York Heart Association on class II or III HF patients with LVEF < 50% suffering from chronic systolic HF. Twenty participants were randomized to probiotic therapy (oral uptake of 1000 mg of *Saccharomyces boulardii*) or placebo for 3 months. The results of the study showed that probiotic uptake in the experimental group resulted in LVEF improvement and reduction in left atrial diameter [45].

### Studies investigating the role of natural phytochemicals in the modulation of the gut microbiota and the management of HF

Research studies are investigating the role of natural phytochemicals in regulating the gut microbiota and metabolites and reducing the risk of HF.

#### Animal studies

Several studies have been conducted on mice to study the effect of phytochemicals on HF patients. However, there was no evidence to support the use of phytochemicals as effective management for HF.

Garlic contains an antibacterial compound called allicin, which reduces TMAO levels [46]. A study was conducted on mice comparing a control group (chow diet group) with the experimental group (allicin plus carnitine diet). The study reported that allicin can protect the host by acting on gut flora via inhibiting TMAO production when carnitine is consumed [47].

Another study was conducted on mice to study the effects of resveratrol (a chemical found in red grapes) on gut microbiota remodeling. This study concluded that resveratrol acts as an anti-inflammatory agent, which reduces the risk of atherosclerosis by shifting gut microbiota composition, lowering plasma TMAO levels, and activating liver bile acid formation [14, 48].

## Future directions

Although several studies have identified a connection between disruptions in gut microbiota, their metabolites, and HF, it remains unclear whether these changes preceded HF or arise because of it. Consequently, more extensive research involving clinical data, dietary factors, and patient history is necessary to investigate the interplay between gut flora composition and HF progression, and to clarify whether a causal relationship exists [49].

Further research is also needed to evaluate and refine microbiota-targeted management strategies for HF. This line of investigation should integrate both mechanistic and applied approaches. On the mechanistic side, advanced sequencing and metabolomic analyses can explore how specific bacterial strains or metabolite profiles modulate cardiac function. On the applied translational side, randomized controlled trials could assess the effectiveness of interventions such as probiotics, prebiotics, antibiotics, or fecal microbiota transplantation (FMT) on clinical outcomes in various HF populations. This work might include (1) identifying optimal microbial strains or metabolic pathways for therapeutic targeting; (2) evaluating the safety and tolerability of microbiota modulation in individuals with HF, including those with comorbidities; and (3) determining how current treatments and lifestyle factors (e.g., diet, physical exercise) can synergize with microbiota-centered treatments.

By bridging fundamental microbiome discoveries with rigorous clinical trials, translational research can clarify whether modifying the gut microbiota can prevent HF onset, slow disease progression, or reduce symptom severity. This approach will help establish evidence-based guidelines for novel microbiota-focused therapies, enhancing the standard of care for patients with HF [49].

## Conclusion

Recent correlation revealed a strong association between gut microbiota and CVDs. Intestinal barrier dysfunction (“leaky gut”) allows bacteria and their metabolites to enter systemic circulation. Among these metabolites, TMAO, LPS, and SCFAs play important roles. Studies have shown that patients suffering from HF tend to have low levels of SCFAs, but elevated levels of TMAO and LPS. TMAO has been associated with endothelial inflammation, foam cell formation, and heightened platelet activity, all of which promote the progression of HF.

HF can be managed conventionally with pharmacological treatments, surgery, and lifestyle modifications. However, ongoing animal studies and clinical trials are exploring microbiota-based strategies such as dietary interventions, antibiotics, fecal microbiota transplantation (FMT), prebiotics, probiotics, and additional approaches to HF management. Research in animal models has already examined antibiotics, FMT, and various microbiota-targeting substances (including natural phytochemicals), while human trials primarily focus on dietary interventions, antibiotics, prebiotics, and probiotics.

Current studies provide evidence linking gut microbiota and heart failure (HF), but the precise pathogenesis and mechanisms of communication remain incompletely understood. Future research should focus on clarifying how specific microbial populations and their metabolites affect HF, potentially revealing new therapeutic targets. Long-term investigations tracking changes in gut microbiota among HF patients could elucidate disease progression, while tailored modulation strategies based on individual microbiome profiles may improve treatment outcomes. Expanding clinical trials that evaluate diverse interventions—such as specialized diets, probiotics, prebiotics, antibiotics, or fecal microbiota transplantation—could help identify effective approaches to gut microbiota modulation in HF management. Integrating advanced sequencing and metabolomic analyses might unearth the bacterial strains or metabolic pathways most relevant to HF, and exploring the gut-heart axis more deeply could uncover biomarkers for earlier diagnosis and risk stratification. Ultimately, understanding the role of the gut microbiota in HF may pave the way for therapies that enhance existing treatments and improve patient well-being.

## Data Availability

No datasets were generated or analysed during the current study.
